# Effectiveness of low power laser in reducing postoperative signs and symptoms after third molar surgery: a triple-blind clinical trial

**DOI:** 10.1590/0103-6440202305413

**Published:** 2023-10-27

**Authors:** Carlos Eduardo Nogueira Nunes, Katlyn Djéssi Silva Andrade, Carlos Aragão Martins, Filipe Nobre Chaves, Denise Hélen Imaculada Pereira de Oliveira, Marcelo Bonifácio da Silva Sampieri

**Affiliations:** 1 Department of Stomatology- School of Odontology, UFC, Federal of University Ceará, Sobral, Ceará, Brazil.

**Keywords:** laser therapy, oral surgery, pain, edema, trismus.

## Abstract

The objective of this research was to evaluate the effectiveness of using LPL (Low power laser) to reduce pain, edema, and trismus after impacted lower third molar extraction. A split-mouth randomized triple-blind clinical trial was conducted at the Federal University of Ceará. For inclusion criteria, it was necessary that the patient presented a clear indication for removal of both lower third molars, in addition to both molars being in similar positions. The third molars (38 and 48) were randomly allocated to the test group that received the LPL application protocol, and to the placebo group that received a simulation of the protocol, making a total sample of 44 surgeries. Patients in the test group used an average of 50% of the amount of analgesics that was used by the placebo group, however, there was a statistically significant difference only on days four and five. Regarding trismus, the test group presented wide mouth openings, both at 48 hours and at 7 days after surgery compared to the placebo group, but without a statistically significant difference. For edema, we noted an equilibrium between the test group and the placebo group, but no measurement obtained a statistically significant difference. The use of LPL presented better pain and trismus indicators after complex extractions. The use of LPL is thus indicated as a complementary therapy to reduce postoperative discomfort caused by complex tooth extractions.

## Resumo

O objetivo desta pesquisa é avaliar a eficácia do uso de LBP (laser de baixa potência) na redução da dor, edema e trismo após exodontia de terceiros molares inferiores impactados. Ensaio clínico randomizado triplo-cego do tipo boca dividida foi realizado na Universidade Federal do Ceará. Para os critérios de inclusão era necessário que o paciente apresentasse indicação clara para a remoção de ambos os terceiros molares inferiores, além da obrigatoriedade de que ambos os molares estivessem em posições semelhantes segundo Winter (1926) e Pell & Gregory (1933). Os terceiros molares (38 e 48) foram alocados aleatoriamente no grupo teste que recebeu o protocolo de aplicação dO LBP e no grupo placebo que recebeu a simulação do protocolo, perfazendo uma amostra total de 44 cirurgias. Os pacientes do grupo teste usaram em média 1/2 da quantidade de analgésicos do que os do grupo placebo, porém, apenas nos dias quatro e cinco houve diferença estatisticamente significativa. Em relação ao trismo, o grupo teste apresentou melhores indicadores tanto em 48 horas quanto em 7 dias após a cirurgia em relação ao grupo placebo, mas sem diferença estatisticamente significante. Em relação ao edema, houve medidas na amostra favoráveis ​​ao grupo teste e medidas favoráveis ao grupo placebo, mas nenhuma das medidas obteve diferença estatisticamente significativa. O uso de LBP mostrou melhores indicadores de dor e trismo após extrações complexas. Portanto, o uso de LBP é indicado como terapia complementar para reduzir morbidades causadas por exodontias complexas.

## Introduction

Over half a century, studies performed on the use of laser therapy have accumulated a considerable amount of evidence on their biological effects in human tissues ^1^.

In dentistry, Masters et al. (1971) first described the use of laser. Laser therapy is currently used in patients for radiation-induced oral mucositis ^2^, recurrent aphthous ulcers ^3^, orofacial pain ^4^ temporomandibular disorders ^4^, and paresthesia repair ^5^ among others.

In dentistry, minor oral surgeries (highly represented by third molar extractions), can cause postoperative discomfort due to muscle spasms with pain, trismus, and edema. All affect the patient´s quality of life ^6^ ^,^ ^7^ ^,^ ^8^ ^,^ ^9^ ^,^ ^10^ ^,^ ^11^ ^,^ ^12^ ^,^ ^13^ ^,^ ^14^ ^,^ ^15^.

Many drugs, such as analgesics and anti-inflammatory drugs are generally effective and used to control postoperative pain ^14^. However, such medications can sometimes bring adverse side effects to the patient, such as gastrointestinal irritation and allergic reactions. The search for alternative methods of postoperative pain control (that avoid side effects) ^6^ ^,^ ^7^ ^,^ ^10^ ^,^ ^12^ ^,^ ^13^ ^,^ ^14^ ^,^ ^15^ ^,^ ^16^ is important. Various methods are being studied, including less traumatic surgical techniques, and laser therapy ^6^ ^,^ ^10^ ^,^ ^12^ ^,^ ^13^ ^,^ ^14^ ^,^ ^15^ ^,^ ^16^.

The lasers used to aid in postoperative healing are low power, presenting no photothermal potential ^9^ ^,^ ^15^ ^,^ ^17^. Studies show that low-power laser (LPL) has the ability to stimulate tissue through photoinduced biochemical interactions which stimulate angiogenesis, vasodilation, and lymphatic drainage while decreasing oxidative stress and edema. LPL can also modulate pain thresholds. All of these effects can influence the healing process ^6^ ^,^ ^8^ ^,^ ^9^ ^,^ ^13^ ^,^ ^14^ ^,^ ^16^ ^,^ ^17^.

Secondary LPL effects include decreasing pro-inflammatory cytokines such as COX-2, interleukin-1b (IL-1b), and IL-6, consequently reducing inflammation, immune response, and pain ^6^ ^,^ ^8^ ^,^ ^9^ ^,^ ^13^ ^,^ ^14^ ^,^ ^16^. In relation to pain, studies indicate that LPL influences serotonin and acetylcholine release, stimulates the production of endorphins, inhibits substances such as bradykinin, and modulates C fiber nerve receptor response, this generates a change in the perception of pain ^15^ ^,^ ^18^. It is noteworthy that all of the benefits presented in the literature as promoted by the use of LPL are also absent of adverse effects ^7^.

Although the subject has been gaining greater attention in recent years, to date there is no single protocol being applied universally. This creates a question as to which of the various protocols present in the literature would deliver the expected benefits of LPL and in the best manner ^15^ ^,^ ^18^. Despite the many application protocols developed (with their distinct results), all of the aforementioned authors have emphasized the need for better-designed randomized clinical trials. By providing more satisfactory samplings, the bias risk would be lower, and definitive conclusions regarding the effects of LPL therapy (in post-surgical third molar extractions), could be defined together with a single effective and validated protocol ^6^ ^,^ ^12^ ^,^ ^15^ ^,^ ^17^ ^,^ ^18^ ^,^ ^19^ ^,^ ^20^ ^,^ ^21^.

In several studies, LPL has presented efficacy in modulating inflammatory processes, and reducing discomfort after surgical procedures. Our research therefore aimed to evaluate the effectiveness of LPL in controlling pain, trismus, and edema after lower third molar extraction. The purpose of this research was to evaluate the effectiveness of using LPL (Low-power laser) to reduce pain, edema, and trismus after impacted lower third molar extraction.

## Methodology

### Study design and sample

After the project was written, (following the full set of ethical principles that govern research on human beings present in the Declaration of Helsinki in 1975, revised in 2013), it was submitted to the research ethics committee of the State University of Vale do Acaraú (UVA), to obtain final approval; Opinion Number 4,351,198. The research was also registered in the Brazilian Clinical Trials Registry (REBEC) at www.ensaiosclinicos.gov.br with the identifier RBR-9k64p2q.

A randomized triple-blind, split-mouth clinical trial was conducted over a 12-month period (September 2020 to September 2021) at the oral surgery clinic of the Federal University of Ceará - Sobral Campus following all of the stipulated biosecurity protocols of the Federal University of Ceará. This protocol included the use of face shields, N95 masks, and surgical pajamas. The authors followed the guideline Consort.Patients aged from 18 to 30 years were selected without regard to gender. For eligibility, it was necessary that the patient presented a clear indication for removal of both lower third molars, and also that both molars be in similar positions according to the Winter (1926) and Pell & Gregory (1933) classifications, and with at least 2/3 of the molar roots having been formed (Nolla - Stage 8).

Participants were excluded from the research for the following criteria: presence of any systemic disease, use of any drug that might interfere with the research data, any allergy or history of adverse reactions to any drug used in the research, the presence of pathologies associated with the lower third molars, or presenting the habits of smoking or consuming alcohol. In addition to these initial criteria: patients who did not follow medication or post-surgical recommendations correctly, did not adequately attend return visits, or presented post-surgical complications (such as alveolitis) were excluded from the sample. However, the excluded patients of this study received proper surgical treatment of third molars following an intention-to-treat approach. The relevance of the split-mouth clinical trial is that the pain experience is compared in the same patient.

In the first consultation, data referring to patient age, sex, and medical and dental history were collected. After explaining the risks and benefits to the potential research participants, those who chose to participate signed an Informed Consent Form (ICF). After data collection and the initial conversations, clinical and radiographic examinations were performed to assess the positions of the third molars, extent of root formation, and tooth impaction. Once the patient selection process was completed, the surgical phase began, with a minimum of 30 days between surgeries on the same patient. We emphasize that all patients were evaluated for active infections, or use of antibiotics, anti-inflammatory drugs, or analgesics for at least 30 days before the procedure. In addition, all patients received the same written post-surgical recommendations, and the same drug therapies at the end of each procedure.

### Sample size

The sampling unit considered for this research consisted of two teeth, with two surgical sites per patient (dental unit). The sample size was selected based on Costa et al. ^22^, a study that demonstrated lower consumption of rescue analgesic medication in a test group using preventive 120 mg etoricoxib when compared to placebos (1.6 and 1.3 vs 4.0 and 2.5). This was deemed necessary to assess 11 surgical sites per study group at a ratio of 1:1 in order to obtain a sample with 80% power, and a 95% confidence interval for the alternative hypothesis (www.openepi.com/SampleSize/SSMean.htm). Oliveira et al. ^23^ replicated these data, with the same success; with a 20% loss of the sample during the study (we therefore estimated 14 surgical sites per group).

### Randomization and blinding

Simple randomization (randomization by envelopes), to decide which region would be operated first, was performed. The designation "X" was used for the lower left third molar (38), and "Y" for the lower right third molar (48). Simple randomization for the groups was also performed using the designation "A1" for the test group that would receive the low-power laser application and “B1” for the placebo group that would receive only the low-power laser placebo.

Randomization was carried out in two stages, (Stage 1) (selecting which tooth would be operated on), and following the surgical procedure (Stage 2) was carried out to select to which group that tooth should be allocated. The patients did not know which group their surgery was allocated to, and the patient was blinded by passing through a simulated laser application (in the same way) as in the test group, using a stopwatch following the same application times and equipment sound simulation (with sound recorded by cell phone) so that the patient would be unaware of any difference between the protocols of the groups. The surgeon was responsible for all surgical procedures and was not informed as to which group each surgery was allocated to, being thus totally blinded during the process. The researcher responsible for the statistics was also blind as to which group the data belonged to and followed the same randomization group designations “X” and “Y” as used in the analysis.

### Surgical procedures and medications

A single surgeon who strictly followed all biosafety protocols performed all surgical procedures. Before the procedure, all patients underwent intraoral antisepsis with 0.12% chlorhexidine gluconate mouthwash for one minute, and extraoral antisepsis with 2% povidone-iodine solution. For each patient, the same surgical protocol was performed in both surgeries in order to standardize the intensity of tissue damage caused in both surgical sites. All patients were anesthetized using a carpule syringe equipped with a long needle and 2 cartridges (4% articaine) of local anesthetic with 1:100,000 epinephrine for truncal anesthesia of the inferior alveolar nerve and infiltrations in the buccal and lingual nerve. A No. 15 blade attached to a No. 3 scalpel handle was used for the initial incision in the distal region of the second molar resting on the retromolar bone, followed by an intra-sucular incision around the gingival sulcus of the second molar. If the surgeon deemed it necessary, a relaxing incision was made, preserving the interdental papilla between the first and second molars in an inferior direction in the buccal sulcus. Continuing, a molt #9 dissector was used for mucoperiosteal detachment. Next, a channel-shaped peripheral osteotomy was performed around the third molar with the aid of a high-speed handpiece coupled to a 702 trunk-conical surgical drill, and if necessary, tooth section was performed at the same time using the same equipment. Tooth removal was performed with the aid of levers and at the end of removal; bone regularization was performed with the aid of a bone file and abundant 0.9% saline solution irrigation. The final suture was performed using 4.0 silk thread and removed one week after the surgical procedure. It is noteworthy that the surgery time (from the beginning of anesthesia to the end of the suture) was always tracked to assess the standardization of procedures.

All patients received orientations on the need for postoperative care in relation to rest, food, and oral hygiene. In addition, the patients were guided to contact the team in case of any complications such as excessive bleeding, exaggerated pain, or signs of infection (fever and suppuration). In addition, all received the postoperative medication nimesulide 100mg, to be taken one tablet every 12 hours for 3 days; and further, only in case of painful symptoms, to ingest a 500mg dipyrone tablet. Now if the pain persisted, they could continue using a 500mg dipyrone tablet every 6 hours, always recording the amount of rescue analgesic ingested. Each patient received a card in which they recorded the amount of rescue medication taken on each postoperative day. In this card was written the postoperative recommendations like - first and second postoperative day: cryotherapy, liquid and cold diet; third day: warm compress and mouthwash with 0.12% chlorhexidine and at the seventh day the suture was removed.

### Laser application protocol

The application of the low-power laser was performed with a Therapy XT (São Carlos/SP, Brazil) device which features both infrared (wavelength of 808nm +/- 10nm) and red (wavelength 660nm +/- 10nm) capacity. Infrared at a wavelength of 808nm +/- 10nm, fiber diameter 600μm, irradiation dose (energy density) of 3J/cm^2^, power at 100 mW, and application time of 40s per point used for research following the parameters reported by Hosseinpour et al.^17^. Using the methodology adapted by Amarillas-Escobar et al. ^25^ Eight application points were determined, four external points (TMJ region, branch region, angle region, and mandible body region), and four internal points (two lateral pterygoid regions, and two surgical wound region). A dentist who did not participate in the surgeries performed the application.

Each patient received two applications of LPL therapy, the first application in the immediate postoperative period, and the second application in the 48-hour postoperative period. In the placebo group, the same periods and times as the test group were respected so that the patient remained blind to the therapy, with the device being taken to the same anatomical regions with a simulated activation sound (made by sound recorded on a cell phone), yet without LPL activation.

### Variables and data collection methods

This research classified variables into the three divisions presented below: Independent or predictive variables (age, sex, position of teeth), dependent variables or outcome (edema, trismus, pain), and covariates or other variables collected in the study (duration of surgery). A single researcher, previously trained in the measurements, collected data and who did not know which group was under consideration (the test group - “A1” or the placebo group - “B1”).

Pain intensity was measured in two ways, the first through the amount of rescue medication used by the patient, and the second through the visual analog scales (VAS) from 0 - 10 (0 as pain free, 10 as worst pain, filled in on the first return visit within 48 hours, and on the second return after 7 days.

The intensity of the edema was measured using anatomical points for the smallest mandibular angle (marking of soft tissue with a permanent marker pen with a thick black tip), and (with a measuring tape) the following anatomical structures: tragus, corner of the eye, nose wing, labial commissure, and chin. Since edema can modify anatomical points, the major limitation of this study maintaining the reference points. The measurement sets were performed in the preoperative period as a comparison mechanism (using the various measurements), and 3 more measurement sets were taken in the immediate postoperative period, in the 48-hour postoperative period, and in the seven-day postoperative period.

Maximum mouth opening was measured using a dry point caliper that started from the incisal edge of the maxillary central incisor to the incisal edge of the mandibular central incisor. This measurement followed the same principle used for edema, with the preoperative mouth opening being measured as a mechanism of comparison to measurements taken in the immediate postoperative period, the 48-hour postoperative period, and in the seven-day postoperative period.

### Statistical analysis

The data obtained in the study were tabulated in the SPSS 23 program and submitted to the Shapiro-Wilk normality test. Quantitative data (duration of surgery, analgesic rescue medication, mouth opening, and facial edema measurements) were expressed as means and standard deviations, and in the Variable Analog Scale (VAS). Nominal qualitative data (radiographic and surgical characteristics) were expressed in absolute and relative frequencies.

The Paired t-test and Wilcoxon test were used to determine statistical significance when comparing paired means between groups (laser vs. placebo), for the respective parametric and nonparametric data.

Analysis between moments for the same group was performed using multiple comparisons of paired averages, in which the parametric data were compared using the paired ANOVA test/Bonferroni post-test, and nonparametric data were compared using the Friedman test/Wilcoxon test with a Bonferroni adjustment. The confidence interval adopted was 95% and the significance level was 5% for all tests. Values of p<0.05 were considered statistically significant.

A descriptive analysis of gender and age variables was performed. Radiographic and surgical characteristics, occurrence of surgical complications, and ingestion of rescue analgesic medication were all evaluated using the McNemar test. A researcher uninvolved in the data collection or surgical procedures performed statistical analysis blindly. Thus, we have the surgeon, patients, and statistician blinded to the LASER and PLACEBO groups, characterizing a triple-blind study. The stated hypotheses were:

H1: Laser is worse than placebo in reducing postoperative pain, trismus, and rescue medication;

H2: Laser is equal to placebo in reducing postoperative pain, trismus, and rescue medication;

## Results

A total of 33 patients were recruited; 7 were excluded from the sample for not meeting the research selection criteria. Of the 26 patients considered eligible for the research, one was excluded from the sample because of postoperative complications, with the need to change the stipulated standard medication, and four were excluded from the sample due to appointment conflicts. The sample unit adopted in this study was the dental unit, with 2 teeth per patient (two surgical sites). The sample size was based on a trial conducted by Costa et al. 2015 ^22^, which demonstrated significant lower rescue analgesic medication (ibuprofen 300mg) consumption in the test group using preemptive etoricoxib 20mg as compared to placebo (mean±SD; 1.6±1.3 *vs* 4.0±2.5; p < 0.05). The OpenEpi online tool (https://www.openepi.com/SampleSize/SSMean.htm) was used for sample size calculation. The calculation was made from the difference of means using Student's T Test. It was judged necessary to include 11 surgical sites per study group (total of 22 sites) at a ratio of 1:1 in order to obtain a sample with 80% power, and a 95% confidence interval for the alternative hypothesis (laser is better than placebo in reducing rescue medication). Further, a loss of 20% of the sample during the study was considered; thus, we estimated 14 surgical sites per group (a total of 28 sites), with a minimum of 14 patients with two low third molars (teeth 38/48).

Thus, at the end of the research and final analysis, 22 patients (44 surgical sites) remained with the relevant characteristics shown in Table 1. InTable 2, we observe that the time used to perform the surgeries did not differ statistically between groups, and thus did not influence pain perception between groups. By the visual numerical scale (VNS), we observe that the placebo group (4.40) presented higher pain perceptions than the test group (3.81), however, without being statistically significant. As to total analgesic intake, we note that the group that received the placebo took twice as many (4.04) rescue analgesics (pills) as the group receiving the laser treatments (2.00). However, even noting this difference, no statistically significant difference was obtained between the groups. It was noted that in the test group, eight patients used no analgesic tablets throughout the postoperative period, this was greater than the placebo group, where only four patients did not feel the need to use rescue medication in the period analyzed. However, when analyzing the postoperative days in isolation, it is possible to show that on every day, the average of number analgesics ingested by the test group was lower than the average of number analgesics ingested by the placebo group. However, this difference was statistically significant only on postoperative days four and five.

**Table 1:** Sociodemographic, radiographic, and surgical characteristics by group (n=22)

Variable	Total (n=22)	Test group (n=22)	Placebo group (n=22)	p-value
Age	22.77 ± 2.54	-	-	-
Gender				-
Female	45.5% (10/22)	-	-	-
Male	55.5% (12/22)	-	-	-
Pell & Gregory Position				
I	22.7% (5/22)	-	-	-
II	77.3% (17/22)	-	-	-
III	0% (0/22)	-	-	-
A	63.6% (14/22)	-	-	-
B	27.3% (6/22)	-	-	-
C	9.1% (2/22)	-	-	-
Winter Position				
Vertical	68.2% (15/22)	-	-	-
Mesioangular	18.2% (4/22)	-	-	-
Horizontal	13.6% (3/22)	-	-	-
Osteotomy	-	81.8% (18/22)	68.2% (15/22)	0,250^a^
Tooth section	-	81.8% (18/22)	68.2% (15/22)	0,125^a^

^a^- McNemar test

**Table 2.** Surgery Time Comparison (STC), Pain Intensity - Variable analog scale (VAS), Total Rescue Analgesic Medication (TRAM), Analgesic Intake (AI), and Daily Analgesic Intake (DAI) (n=22)

Variable	Test group (n=22)		Placebo group (n=22)		p-value
	Average	CI	Average	CI	
STC	21.09 (±7.67)	17.68 - 24.49	23.45 (±10.16)	18.95 - 27.96	0.056^to^
VAS	3.81 (±2.75)	2.59 ± 5.03	4.40 (± 2.80)	3.16 ± 5.65	0.324^b^
TRAM	2.00 (±2.04)	1.09 - 2.90	4.04 (±4.58)	2.01 - 6.07	0,453^c^
AI (yes/no)	(15/8)		(18/4)		0.488^c^
DAI					
Day 1	0.77 (±0.92)	0.36 - 1.18	1.00 (±0.87)	0.61 - 1.39	0.379^b^
Day 2	0.55 (±0.80)	0.19 - 0.90	0.73 (±0.98)	0.29 - 1.16	0.432^b^
Day 3	0.41 (±0.66)	0.11 - 0.70	0.55 (±1.01)	0.10 - 0.99	0.490^b^
Day 4	0.09 (± 2.94)	0.04 - 0.22	0.59 (±0.95)	0.17 - 1.02	0.035^b*^
Day 5	0	0	0.41 (±0.79)	0.06 - 0.76	0.034^b*^
Day 6	0.14 (±0.35)	0.02 - 0.29	0.36 (±0.72)	0.04 - 0.69	0.160^b^
Day 7	0.05 (±0.21)	0.05 - 0.14	0.32 (±0.64)	0.03 - 0.60	0.084^b^

^a^- Paired Test T;^b^- Wilcoxon Test;^c^ - McNemar Test; * p<0.05

InTable 3, we compare preoperative maximum mouth opening (MMO) with the postoperative period. When comparing the initial mouth opening with the immediate postoperative period, we noticed a slight advantage for the placebo group, which presented a reduction of 5.72 mm in MMO, as compared to the test group, which presented a reduction of 6.87 mm in the MMO; a difference of 1.15mm between groups. When comparing the initial MMO at the 48-hour postoperative period, we noticed an advantage for the group that received laser and saw a reduction in MMO of 9.41mm compared to the group that received placebo, which saw a reduction in MMO of 13.31mm, a 3.9mm difference between the groups. When then comparing the initial MMO at the 7-day postoperative period, we noticed an advantage for the group that received laser, which presented a reduction in MMO of 5.50mm compared to the group that received the placebo, which presented a reduction in MMO of 9.22mm, a difference of 3.72mm between the groups. We emphasize that although the test group presented better results compared to the placebo group in the 48-hour and 7-day postoperative periods, there was no statistical significance between them.

**Table 3:** Comparison of maximum mouth opening (mm) in different evaluation periods (mean ± SD). (n=22)

Time course	Test group (n=22)		Placebo group (n=22)		p-value
	Mean ±SD	95% CI	Mean ±SD	95% CI	
Pre-Operative	43.59 ± 6.37	40.76 - 46.41	44.90 ± 6.20	42.15 - 47.65	0.124^to^
Post-Op Immediate	36.72 ± 10.08^#^	32.25 - 41.19	39.18 ± 9.04^#^	35.17 - 43.19	0.221^to^
After 48h	34.18 ± 9.97^#^	29.75 - 38.60	31.59 ± 9.97^#&^	27.16 - 36.01	0.242^to^
Post-Op 7 days	38.09 ± 7.84^#^	34.61 - 41.57	35.68 ± 8.34^#@^	31.98 - 39.37	0.163^to^
p-value	0.000^y*^		0.000^y*^		

^a^- Paired Test T;^b^- Wilcoxon Test;^y^- ANOVA/Bonferroni; * - p<0.05/^#^- p<0.05 with respect to measure/ MMO 1 (two-way ANOVA for repeated samples with Bonferroni adjustment)/& - p<0.05 with respect to measure MMO 2 (two-way ANOVA for repeated samples with Bonferroni adjustment)/@ - p<0.05 in relation to the MMO 3 measure (two-way ANOVA for repeated samples with Bonferroni adjustment)

InTable 4, we analyze the influence of laser in controlling postoperative edema; measurements of distances between fixed facial points were performed for the periods noted. As is well established in the literature, the measurements taken at 48 hours in both groups presented the highest averages both in the test group and in the placebo group. Comparing both groups, we noted an equilibrium between the groups, and there was no statistically significant difference between the groups regarding facial edema.

**Table 4:** Comparison between fixed facial measurements in different periods evaluated

Facial points	Time Period	Test side (n=22)	Placebo side (n=22)
AM - M	Initial	10.70 (± 2.24)	11.16 (± 0.89)
	1st P	11.35 (± 0.69)^@^	11.39 (± 0.63)
	48h	11.55 (± 0.70)^@#^	11.63 (± 0.67)^@#^
	7 days	11.37(±0.69)^@&^	11.32 (± 0.67)^&^
p-value		0.000z*	0.000z*
AM - CL	Initial	9.17 (± 0.86)	9.29 (± 0.66)
	1st P	9.37 (± 0.68)	9.28 (± 0.67)
	48h	9.60 (± 0.76)^A^	9.56 (± 0.66)^@#^
	7 days	9.36 (± 0.66)^C^	9.41 (± 0.62)^&^
p-value		0.009^y^*	0.001^z^*
AM - NA	Initial	11.34 (± 1.02)	11.37 (± 0.67)
	1st P	11.70 (± 0.77)^A^	11.49 (± 0.72)
	48h	11.64 (± 0.82)	11.59 (± 0.72)^A^
	7 days	11.51 (± 0.82)	11.50 (± 0.62)
p-value		0.036^y^*	0.053^y^
AM - CO	Initial	10.49 (± 0.71)	10.40 (± 0.75)
	1st P	10.63 (± 0.68)^A^	10.51 (± 0.76)^A^
	48h	10.66 (± 0.76)	10.48 (± 0.75)
	7 days	10.56 (± 0.70)	10.43 (± 0.76)
p-value		0.002^y^*	0.021^y^
AM - TG	Initial	5.90 (± 0.51)	5.79 (± 0.55)
	1st P	5.98 (± 0.54)	5.95 (± 0.60)^@^
	48h	6.02 (± 0.69)	5.95 (± 0.58)^@^
	7 days	6.00 (± 0.58)	5.95 (± 0.62)^@^
p-value		0.162^y^	0.000^z^*

^a^- Paired Test T; b - Wilcoxon Test; y - ANOVA /Bonferroni; z - Friedman/Wilcoxon/Bonferroni * p<0.05

^@^- p<0.05 in relation to T1 (Friedman with Wilcoxon post-test and Bonferroni adjustment)

^#^- p<0.05 in relation to T2 (Friedman with Wilcoxon post-test and Bonferroni adjustment)

^&^- p<0.05 in relation to T3 (Friedman with Wilcoxon post-test and Bonferroni adjustment)

^A^- p<0.05 in relation to T1 (ANOVA for repeated samples with Bonferroni adjustment)

^B^- p<0.05 in relation to T2 (ANOVA for repeated samples with Bonferroni adjustment)

^C^- p<0.05 in relation to T3 (two-way ANOVA for repeated samples with Bonferroni adjustment)

## Discussion

The LPL therapies have gained greater notoriety in recent years, but so far there has been protocol standardization applied by all authors, raising the question of which of the protocols presents the expected results for the use of LPL ^15^ ^,^ ^18^.

The first study on this topic found in the literature was Roynesdal et al. ^19^, where the protocol involved a Biophoton Laser (Roenvig Dental, Denmark), with power at 40 mW, a wavelength of 820 nm, and an energy density of 6J/cm^2^. The use of LPL provided no benefits in relation to post-operative edema, trismus, or pain. Further, the authors did not specify which regions the points were placed ^19^.

Aras et al. ^21^, using the Ga-Al-As diode laser protocol (Doctor Smile erbium and laser diode; Lambda Scientifica Srl, Vicenza, Italy), at 808nm, power at 100 mW, application time of 120 seconds, and energy density of 12J/cm^2^ in intraoral surgical wounds, and in the extraoral masseter insertion region, obtained significant (good) results for the use of LPL in the reduction of both edema and trismus after third molar extractions.

More recently, Santos et al. ^20^, adopted an LPL protocol with five application points (two buccal, two lingual and one in the surgical wound) - all intraoral, using MM Optics Twin Flex Evolution (Opto-Electronic Equipment, São Carlos, Brazil), with infrared radiation emission (at 780 nm, power at 70 mW, and energy density at 52.5J/cm2). The irradiation time was 30 seconds per point. When this LPL equipment was used with this protocol, the group found satisfactory results for pain reduction when compared to the placebo.

Systematic reviews on the subject ^15^ ^,^ ^17^ used the following inclusion parameters as research criteria: that studies be randomized clinical trials comparing LPL with placebo, and that in addition to primary predictive variables, edema, trismus, and postoperative pain be measured on the second and seventh post-operation days.

A single surgeon who was unaware of which group the procedure would fit performed the surgeries in this research. The patient was also unaware of whether or not he was receiving therapy with LPL, because he was receiving a simulation with the same sound stimuli as in the real LPL procedure. In addition, the statistician responsible for the analyses received the data tabulated in codes (Test group = X, Placebo group = Y). In view of these precautions, this study produced a transparent methodological approach (without permitting data or interference from the researchers involved), which is fitting for a high-quality, randomized, and a triple-blind clinical trial, with a low risk of bias. The approach used is thus legitimate for evaluating the different post-operative pain protocols.

Domah et al.^15^, in a systematic review that included all LPL studies (diode lasers, infrared lasers, helium-neon lasers, and gallium-aluminum-arsenic lasers), considered using wavelengths from 600 to 1000 nm, output power from 10 to 500 mW, energy densities between 3 and 12 J/cm^2^,^ ^ and an application time of between 15 and 180 seconds, and found reductions in edema, trismus, and postoperative pain with the use of LPL, however, only the edema results were considered statistically significant.

In another systematic review, Hosseinpour et al.^17^ evaluated which LPL specifications were used when the results were positive for reducing postoperative discomfort. It was demonstrated that studies using wavelengths ranging from 650 to 980 nm, power between 4 and 300mW, and energy densities between 3 and 85.7 J/cm^2^were effective in reducing post-operative pain. It was also evidenced that studies using wavelengths between 660 and 910 nm, power between 4-500mW, and energy densities between 2-480 J/cm^2^ were effective in reducing facial edema ^17^. It was shown as well that wavelengths between 660 and 980 nm, power between 4 and 300mW, and energy densities between 4-106 J/cm^2^were effective in reducing trismus ^17^.

The LPL in our research was applied using a device called Therapy XT from the company DMC (São Carlos/SP, Brazil), with infrared technology, at a wavelength of 808nm +/- 10nm, fiber diameter of 600μm, an irradiation dosage of 3J/cm^2^, power at 100 mW, and an application time of 40s per point, in accordance with the parameters described by Hosseinpour et al.^17^, and using the methodology adapted by Amarillas-Escobar et al. ^25^.

As to postoperative pain, a recent systematic review ^17^ revealed that in most of the studies included, 60% found positive results for pain reduction in the first few days after their surgical procedure. It is noted that certain studies lacked significant results, yet these results corroborate the findings of our research, where subjective (VAS) and objective parameters (days four and five postoperative analgesic rescue medications), presented significantly better results in the test group than in the placebo group.

A split-mouth clinical trial ^20^ carried out with 32 patients and 64 surgical procedures found significant results for postoperative pain control with the use of LPL, corroborating the results of our study. The sample of the study performed by Santos et al. (2020), was 64 surgeries, being larger than in this study, where 44 surgeries were performed. The study divided the sample into 3 groups, where in-group 1, only standard postoperative guidelines were performed, in-group 2, LPL was applied, and in-group 3, LPL application was simulated. A statistically significant result was observed for pain reduction in all parameters when comparing group 2 to group 3 ^14^. Further, in 2016, a study by Alan et al. ^11^, where 30 surgeries were performed, presented better results in relation to pain in the group that received LPL, than in the placebo group. Yet, only on the seventh postoperative day was this difference considered statistically significant.

Finally, although a statistically significant difference was evidenced only in a portion of the parameters studied, the lower use of analgesics by patients who received LPL applications is important; it implies more patient comfort, less spending on medication, and a lower risk of negative drug interactions during the postoperative period. The time spent to perform the study was 1 year (due to the COVID-19 pandemic). The costs involving laser therapy device, surgical instruments and dental materials were in the order of U$ 1064,00.

With regard to mouth opening, the test group receiving LPL application presented values ​​closer to the original mouth opening measurements (during the postoperative periods of 48 hours and 7 days) than the placebo group. Although these data are not statistically significant, the explanation for the greater mouth opening in the test group may be due to LPL´s analgesic and anti-inflammatory activity. LPL was applied to the muscles responsible for opening the mouth. A study using a methodology similar to ours and with a smaller sample (30 surgeries), carried out in 2016 by Alan and collaborators ^11^ found better mouth-opening results in the test group than in the placebo group, this for the same 48 hour and 7 day periods (post-procedure), yet without any statistically significant difference.

Edema measurements varied between favoring the placebo group to favoring the test group. In any case, the data were not statistically significant. As found in previous studies ^11^ ^,^ ^13^, it was expected that there would be greater edema reductions in the test group compared to the placebo group at all craniometrics points. However, this effect of reducing edema seems more subtle, since although the results were better towards reducing edema, they were not statistically significant in any period evaluated ^11^ ^,^ ^13^. It is important to note that posing a “standardized method” of quantifying edema is somewhat controversial since in the literature recent studies use both techno-photographic quantification of edema ^11^ ^,^ ^14^, and anatomical points and distances in methods quite similar to those used in this study ^24^ ^,^ ^26^ ^,^ ^27^.

Despite the absence of a standardized protocol for applying LPL, we cannot rule out the effects of LPL in reducing postoperative discomfort. We observed a considerable and statistically significant decrease in postoperative rescue medication use by the test group on postoperative days four and five, in addition to the improvement in mouth opening on the second and seventh postoperative days. Several studies have already corroborated our findings ^11^ ^,^ ^13^ ^,^ ^14^ ^,^ ^20^. It is worth mentioning that LPL has been defended in recent studies as safe and applicable as an auxiliary therapy to heal surgical wounds ^13^. To provide the patient with a more comfortable, safe, and cost-effective postoperative experience, reducing postoperative discomfort is extremely important.

In this clinical trial, as compared to the placebo group, reductions in pain (statistically significant on days four and five) and trismus were found for the group that received LPL at intraoral and extraoral points. In addition, the use of LPL as a complementary therapy is considered safe and effective in helping to reduce pain and trismus after complex third molar extractions, and its use is indicated.

The laser was effective in reducing pain on the fourth and fifth postoperative days, a period in which the patient feels the greatest discomfort. Other studies also show the effectiveness of the laser in reducing pain in the postoperative period, so we believe that its application in the postoperative period in third-molar surgeries is important. The biggest limitation of our study was the small sample size, due to the COVID-19 pandemic, which made access to patients difficult. More triple-blind, randomized, split-mouth clinical trials are needed to provide better indications for LPL use.

## References

[1] Cronshaw M, Parker S, Anagnostaki E, Mylona V, Lynch E, Grootveld M (2020). Photobiomodulation and oral mucositis: A systematic review. Dent J.

[2] Sălăgean T, Pop ID, Todea D, Bordea IR, Dalvi S, Benedicenti S (2020). Photobiomodulation therapy in oral mucositis and potentially malignant oral lesions: A therapy towards the future. Cancers.

[3] Suter VGA, Sjölund S, Bornstein MM (2017). Effect of laser on pain relief and wound healing of recurrent aphthous stomatitis: a systematic review. Lasers Med Sci.

[4] Zokaee H, Zahmati AHA, Mojrian N, Boostani A, Vaghari M (2018). Efficacy of Low- Level Laser Therapy on Orofacial Pain: A Literature Review. Adv Hum Biol.

[5] Bittencourt MAV, Paranhos LR, Martins-Filho PRS (2017). Low-level laser therapy for treatment of neurosensory disorders after orthognathic surgery: A systematic review of randomized clinical trials. Med Oral Patol Oral Cir Bucal.

[6] He WL, Yu FY, Li CJ, Pan J, Zhuang R, Duan PJ (2015). A systematic review and meta-analysis on the efficacy of low-level laser therapy in the management of complication after mandibular third molar surgery. Lasers Med Sci.

[7] Fabre H. S. C, Navarro R. L., Paula V.P, Oltramari-Navarro R. F. O, Deise A. A. P. O, Rodrigo A. C. A, Karen B. P. F, Nels on F (2015). Anti-inflammatory and analgesic effects of low-level laser therapy on the postoperative healing process. J. Phys. Ther. Sci.

[8] Sierra SO, Deana AM, Bussadori SK, Da Mota ACC, Motta LJ, Ferrari RAM (2015). Effect of low-intensity laser treatment on pain after extraction of impacted mandibular third molars: A randomised, controlled, clinical trial. Br J Oral Maxillofac Surg.

[9] Pol R, Ruggiero T, Gallesio G, Riso M, Bergamasco L, Mortellaro C (2016). Efficacy of anti-inflammatory and analgesic of superpulsed low level laser therapy after impacted mandibular third molars extractions.. J Craniofac Surg.

[10] Kahraman SA, Cetiner S, Strauss RA (2017). The Effects of Transcutaneous and Intraoral Low-Level Laser Therapy after Extraction of Lower Third Molars: A Randomized Single Blind, Placebo Controlled Dual-Center Study. Photomed Laser Surg.

[11] Alan H, Yolcu Ü, Koparal M, Özgür C, Öztürk SA, Malkoç S (2016). Evaluation of the effects of the low-level laser therapy on swelling, pain, and trismus after removal of impacted lower third molar. Head Face Med.

[12] Dawdy J, Halladay J, Carrasco-Labra A, Araya I, Yanine N, Brignardello-Petersen R (2017). Efficacy of adjuvant laser therapy in reducing postsurgical complications after the removal of impacted mandibular third molars: A systematic review update and meta-analysis. J Am Dent Assoc.

[13] Choung HW, Lee SH, Ham AR, Lee NR, Kim B, Pang KM (2019). Effectiveness of low-level laser therapy with a 915 nm wavelength diode laser on the healing of intraoral mucosal wound: An animal study and a double-blind randomized clinical trial. Med.

[14] F Asutay AO (2018). H Alan2 MK. Therapy on Facial Swelling after Lower Third Molar Surgery : A Randomized, Placebo-Controlled Study. Niger J Clin Pract.

[15] Domah F, Shah R, Nurmatov UB, Tagiyeva N (2021). The Use of Low-Level Laser Therapy to Reduce Postoperative Morbidity After Third Molar Surgery: A Systematic Review and Meta-Analysis. J Oral Maxillofac Surg.

[16] Dostalova T, Kroulikova V, Podzimek S, Jelinková H (2017). Low-Level Laser Therapy after Wisdom Teeth Surgery: Evaluation of Immunologic Markers (Secretory Immunoglobulin A and Lysozyme Levels) and Thermographic Examination: Placebo Controlled Study. Photomed Laser Surg.

[17] Hosseinpour S, Tunér J, Fekrazad R (2019). Photobiomodulation in Oral Surgery: A Review. Photobiomodul Photomed Laser Surg.

[18] Brignardello-Petersen R, Carrasco-Labra A, Araya I, Yanine N, Beyene J, Shah PS (2012). Is adjuvant laser therapy effective for preventing pain, swelling, and trismus after surgical removal of impacted mandibular third molars? A systematic review and meta-analysis. J Oral Maxillofac Surg.

[19] Roynesdal AK, Björnland T, Barkvoll P, Haanaes HR (1993). The effect of soft-laser application on postoperative pain and swelling. A double-blind, crossover study. Int J Oral Maxillofac Surg.

[20] Santos PL, Marotto AP, Zatta da Silva T, Bottura MP, Valencise M, Marques DO (2020). Is Low-Level Laser Therapy Effective for Pain Control After the Surgical Removal of Unerupted Third Molars? A Randomized Trial. J Oral Maxillofac Surg.

[21] Aras MH, Güngörmüş M (2009). The effect of low-level laser therapy on trismus and facial swelling following surgical extraction of a lower third molar. Photomed Laser Surg.

[22] Costa FWG, Soares ECS, Esses DFS (2015). A split-mouth, randomized, triple-blind, placebo-controlled study to analyze the preemptive effect of etoricoxib 120mg on inflammatory events following removal of unerupted mandibular third molars. Int JOral Maxillofacial Surg.

[23] Oliveira EM, Oliveira VB, Araújo LK, Lopes TS, Rego RO, Sampieri MBDS. (2021). Anti-Inflammatory Effectiveness of Oral Dexamethasone 4 mg on Mandibular Third Molar Surgeries: A Split-Mouth Randomized Clinical Trial. J Oral Maxillofac Surg.

[24] Sampaio-Filho H, Bussadori SK, Gonçalves MLL, Da Silva DDFT, Borsatto MC, Tortamano IP (2018). Low-level laser treatment applied at auriculotherapy points to reduce postoperative pain in third molar surgery: A randomized, controlled, single-blinded study. PLoS One.

[25] Amarillas-Escobar ED, Toranzo-Fernández JM, Martínez-Rider R, Noyola-Frías MA, Hidalgo-Hurtado JA, Serna VM, Gordillo-Moscoso A, Pozos-Guillén AJ (2010). Use of therapeutic laser after surgical removal of impacted lower third molars. J Oral Maxillofac Surg.

[26] Sigaroodi AK, Motevasseli S, Maleki D, Maleki D, Fard RS (2023). Low-level laser and management of common complications after the mandibular third molar surgery: A double-blind randomized clinical trial. Dent Res J.

[27] Fraga RS, Antunes LAA, Fialho WLS (2020). Do Antimicrobial Photodynamic Therapy and Low-Level Laser Therapy Minimize Postoperative Pain and Edema After Molar Extraction?. J Oral Maxillofac Surg.

